# Phylogenetic Analysis of the Kinesin Superfamily from *Physcomitrella*

**DOI:** 10.3389/fpls.2012.00230

**Published:** 2012-10-16

**Authors:** Zhiyuan Shen, Angelo R. Collatos, Jeffrey P. Bibeau, Fabienne Furt, Luis Vidali

**Affiliations:** ^1^Department of Biology and Biotechnology, Worcester Polytechnic InstituteWorcester, MA, USA

**Keywords:** phylogenetic analysis, kinesin, microtubule, moss, phragmoplast, gene knockout, intracellular motility, intracellular transport

## Abstract

Kinesins are an ancient superfamily of microtubule dependent motors. They participate in an extensive and diverse list of essential cellular functions, including mitosis, cytokinesis, cell polarization, cell elongation, flagellar development, and intracellular transport. Based on phylogenetic relationships, the kinesin superfamily has been subdivided into 14 families, which are represented in most eukaryotic phyla. The functions of these families are sometimes conserved between species, but important variations in function across species have been observed. Plants possess most kinesin families including a few plant specific families. With the availability of an ever increasing number of genome sequences from plants, it is important to document the complete complement of kinesins present in a given organism. This will help develop a molecular framework to explore the function of each family using genetics, biochemistry, and cell biology. The moss *Physcomitrella patens* has emerged as a powerful model organism to study gene function in plants, which makes it a key candidate to explore complex gene families, such as the kinesin superfamily. Here we report a detailed phylogenetic characterization of the 71 kinesins of the kinesin superfamily in *Physcomitrella*. We found a remarkable conservation of families and subfamily classes with *Arabidopsis*, which is important for future comparative analysis of function. Some of the families, such as kinesins 14s are composed of fewer members in moss, while other families, such as the kinesin 12s are greatly expanded. To improve the comparison between species, and to simplify communication between research groups, we propose a classification of subfamilies based on our phylogenetic analysis.

## Introduction

Kinesins are a superfamily of microtubule (MT) dependent motors that are present in all eukaryotes (Richardson et al., 2006). The critical importance for cell function of this superfamily is highlighted by its existence and diversification in the last common ancestor of plants, animals, and fungi. The members of the various families of kinesins perform a multitude of functions, but they are all related by their conserved motor domain (Miki et al., 2005).

The kinesin motor domain, or head, comprises approximately 360 amino acids, and contains the ATPase and MT binding activities. The motor domain can be located either at the C-terminus, N-terminus, or in the middle of the molecule. In addition to the motor domain, most kinesins have a neck region that contains family specific features, a coiled coil region that is important for dimerization, and a tail region that is thought to bind to specific cargo. The directionality of kinesin varies between families, and is sometimes correlated with the position of the motor. The directionality of all the kinesin is not known, but in general, members of the kinesin 1 and 2 families travel to the plus end of MTs, while members of the kinesin 14 family travel toward the minus ends.

Because of the large size of the kinesin superfamily, it has been important to unify the nomenclature across phyla to allow comparative analyses of function. A standardized nomenclature was proposed by a special interest subgroup of the American Society of Cell Biology (ASCB), which has been broadly adopted (Lawrence et al., 2004). This nomenclature separates all major kinesins into 14 families. Kinesins that do not belong to any of these families are considered orphans, but most kinesins identified can easily be assigned to a specific family. Together with the development of high throughput and next generation genomic sequencing, important efforts have taken place to use phylogenetic analysis and classification in diverse species ranging from unicellular to multicellular organisms to explore the large set of functions fulfilled by kinesins (Miki et al., 2005; Richardson et al., 2006).

In plants, kinesins have been implicated in a variety of cellular processes, including intracellular transport, spindle assembly, chromosome motility, phragmoplast assembly, MAP kinase regulation, and MT stability (Vale, 2003; Lee and Liu, 2004; Cai and Cresti, 2010; Zhu and Dixit, 2011a). Plants contain almost all the kinesin families, including kinesins important for flagellar development that are only present in plants with motile sperm, such as ferns and mosses (this study). Occasionally, the function of some members of a family do not appear to be conserved with its animal and fungal counterparts, and plants also contain specific kinesin families (Richardson et al., 2006).

The kinesin content has been determined in various plants. Reddy and co-workers identified 61, 52, 41, and 45 kinesins in *Arabidopsis*, poplar, and two cultivars of rice, respectively (Richardson et al., 2006), while the red algae *Cyanidioschyzon merolae* contains only five kinesins and the green algae *Chlamydomonas reinhardtii* 23 (Reddy and Day, 2001; Richardson et al., 2006). However, the full set of kinesins in basal land plants has yet to be investigated.

The moss *Physcomitrella patens* is a simple plant model organism that allows precise genetic manipulations and provides easy access to cells for high resolution microscopy (Cove, 2005). This makes it an ideal model system to study the participation of the MT cytoskeleton in many different processes. Surprisingly, only two kinesins, KINID1a and KINID1b, hereafter called Pp-KinesinOrph-IIa and Pp-KinesinOrph-IIb, have been studied in *Physcomitrella* and have been shown to be essential for the generation of interdigitated antiparallel MT in the phragmoplast (Hiwatashi et al., 2008). This highlights the need to have a complete inventory of the multitude of kinesins present in this organism to help perform future functional analysis. With an available genome sequence (Rensing et al., 2008) it is now possible to document all the kinesins present in this organism. In the present work, we perform a phylogenetic analysis of 71 kinesins from *Physcomitrella*, identified from their conserved motor domain.

## Materials and Methods

Kinesin motor domain sequences were identified by BLAST against the cosmoss.org version 1.6; the 6th annotation of the *Physcomitrella* first genome assembly (Rensing et al., 2008), and the protein sequences were identified from predicted gene models. A total of 71 sequences were identified (Table 1), the head domain was extracted from the sequences by alignment comparison with a template based on the kinesin 1 head domain (Uniprot: P33176).

**Table 1:** **Kinesin families and classes in *Physcomitrella patens***.

Kinesin family	Gene name	Gene ID (Phypa_#)
Kinesin ARK (*n* = 5)	Pp-KinesinARK-a	455498
	Pp-KinesinARK-b	453488
	Pp-KinesinARK-c	425827
	Pp-KinesinARK-d	427907
	Pp-KinesinARK-LIKE	446331
Kinesin 2 (*n* = 1)	Pp-Kinesin02	425592
Kinesin 4 (*n* = 8)	Pp-Kinesin04-Ia	437833
	Pp-Kinesin04-Ib	438737
	Pp-Kinesin04-Ic	432365
	Pp-Kinesin04-Id	453193
	Pp-Kinesin04-Ie	441211
	Pp-Kinesin04-IIa	447296
	Pp-Kinesin04-IIb	433281
	Pp-Kinesin04-IIc	446183
Kinesin 5 (*n* = 4)	Pp-Kinesin05-a	457162
	Pp-Kinesin05-b	447260
	Pp-Kinesin05-c	425536
	Pp-Kinesin05-d	423604
Kinesin 7 (*n* = 7)	Pp-Kinesin07-Ia	447411
	Pp-Kinesin07-Ib	437231
	Pp-Kinesin07-IIa	458197
	Pp-Kinesin07-IIb	432536
	Pp-Kinesin07-IIc	454208
	Pp-Kinesin07-III	426030
	Pp-Kinesin07-IV	452429
Kinesin 8 (*n* = 3)	Pp-Kinesin08-Ia	453903
	Pp-Kinesin08-Ib	424121
	Pp-Kinesin08-Ic	458481
Kinesin 9 (*n* = 3)	Pp-Kinesin09-Ia	458410
	Pp-Kinesin09-Ib	425498
	Pp-Kinesin09-Ic	428375
Kinesin 12 (*n* = 18)	Pp-Kinesin12-Ia	444072
	Pp-Kinesin12-Ib	440218
	Pp-Kinesin12-Ic	442090
	Pp-Kinesin12-Id	437562
	Pp-Kinesin12-Ie	434464
	Pp-Kinesin12-If	432190
	Pp-Kinesin12-Ig	432169
	Pp-Kinesin12-Ih	454564
	Pp-Kinesin12-Ii	432906
	Pp-Kinesin12-Ij	445541
	Pp-Kinesin12-Ik	422406
	Pp-Kinesin12-Il	431567
	Pp-Kinesin12-Im	453302
	Pp-Kinesin12-In	426336
	Pp-Kinesin12-Io	437642
	Pp-Kinesin12-IIa	422514
	Pp-Kinesin12-IIb	422285
	Pp-Kinesin12-IIc	440124
Kinesin 13 (*n* = 3)	Pp-Kinesin13-Ia	427794
	Pp-Kinesin13-Ib	438664
	Pp-Kinesin13-Ic	456175
Kinesin 14 (*n* = 15)	Pp-Kinesin14-Ia	439730
	Pp-Kinesin14-Ib	438782
	Pp-Kinesin14-IIa	430601
	Pp-Kinesin14-IIb	439319
	Pp-Kinesin14-IIc	436987
	Pp-Kinesin14-IId	441550
	Pp-Kinesin14-IIIa	459874
	Pp-Kinesin14-IIIb	424496
	Pp-Kinesin14-IV	435249
	Pp-Kinesin14-Va	437825
	Pp-Kinesin14-Vb	435597
	Pp-Kinesin14-VIa	439249
	Pp-Kinesin14-VIb	450599
	Pp-Kinesin14-VIc	428061
	Pp-Kinesin14-VId	458819
Kinesin orphans (*n* = 9)	Pp-KinesinOrph-Ia	457477
	Pp-KinesinOrph-Ib	453299
	Pp-KinesinOrph-IIa	436446
	Pp-KinesinOrph-IIb	430757
	Pp-KinesinOrph-III	441202
	Pp-KinesinOrph-IVa	431083
	Pp-KinesinOrph-IVb	451243
	Pp-KinesinOrph-IVc	437822
	Pp-KinesinOrph-IVd	453297

*The gene ID can be used to retrieve genes from www.cosmoss.org. From the pull down menu under “genome” in the homepage, select “sequence retrieval” and type in the format of “Phypa_#,” for example, “Phypa_453297.” This should lead you to the gene that the specific Phypa number designates*.

For phylogenetic comparison, the sequences were imported into Vector NTI Advance 11.5.1 (Invitrogen), and an alignment was generated using its AlignX program. The basic algorithm from AlignX is ClustalW; we maintained the default parameters as follows: gap opening penalty: 10, gap extension penalty: 0.05, gap separation penalty range: 8, percent identity for alignment delay: 40. The alignment was further improved by identifying the members of each family of kinesins using the fast neighbor distance-based algorithm from AlignX, and aligning the groups separately. The assignment to specific families was very consistent for the majority of the sequences identified. This was done to remove possible minor errors (Figure S1 in Supplementary Material) in the gene models, which were in general present at a low frequency. For the final alignment, the protein sequences of all the motor domains from all the families were used and the sequence for the globular tail domain of *Physcomitrella’s* myosin XIa (Uniprot: D6R266) was used as an outgroup.

Once a satisfactory alignment was completed, the alignment file was imported to Geneious (Biomatters Ltd.), where a tree was constructed using the plugin PhyML that applies the Maximum Likelihood method (Guindon et al., 2010). We maintained the default parameters as follows: substitution model: LG, proportion of invariable sites: 0-fixed, number of substitution rate categories: 1, no optimization, and a 1000 bootstrap resampling value. To help identify the various family groups, a representative member from human and all *S. pombe* and *Saccharomyces cerevisiae* kinesins were included in the alignment. In addition, the complete collection of the *Arabidopsis* kinesins was included for comparison.

Our preliminary trees constructed with the neighbor joining algorithm available in AlineX from Vector NTI Advance resulted in similar topologies for most classes. Furthermore, in the majority of the families, the human representative sequence is present, providing good support to our alignment and tree building strategy. In the tree that we present here, only nodes showing more than 50% bootstrap support are indicated, and the bootstrap support is shown.

We have used a nomenclature based, when available, on the kinesin family name designated by a number (Lawrence et al., 2004), followed by a class number (indicated by roman numerals; Table 1). To identify individual members of the classes we used letters in the case of *Physcomitrella* and numbers in the case of *Arabidopsis* in order to avoid possible future confusion when the classes are monophyletic and no clear orthologs are present between species.

## Results and Discussion

In the following sections we report our findings on the number and class of kinesins for each subfamily and when possible discuss their predicted function based on comparison to other similar kinesins. A clear kinesin 1 member is not present in *Physcomitrella*, but interestingly kinesin 1 members are present in *Arabidopsis* and other seed plants (Richardson et al., 2006; Zhu and Dixit, 2011a). Because of their similarity with kinesin 1s, we decided to start our report with the Armadillo Repeat containing Kinesins (ARK), and we decided to not include them in the orphan section since they are well conserved across plants and can be clearly identified as a separate group. We were also not able to unequivocally assign moss proteins to families 3, 6, 10, and 11; a more detailed discussion about this is presented in the last section concerning orphan kinesins.

### ARK kinesins

This kinesin family is characterized and classified by the armadillo repeat motifs found within the protein’s C-terminal domain. Armadillo repeats are comprised of a repeating sequence of forty-two amino acids (Coates, 2003). This sequence contains three alpha helices; upon repetition these helices form a right handed super helix (Coates, 2003). Typically, these repeats are associated with cell signaling and the cytoskeleton. In *Arabidopsis*, it has been speculated that the armadillo regions bind to target proteins to aid in their MT based transport (Coates, 2003). Additionally, loss of function analysis of armadillo kinesins in *Arabidopsis* root hairs suggests that these proteins may play a significant role in actin and MT organization during polarized cell growth (Yang et al., 2007; Sakai et al., 2008).

**Figure 1:**
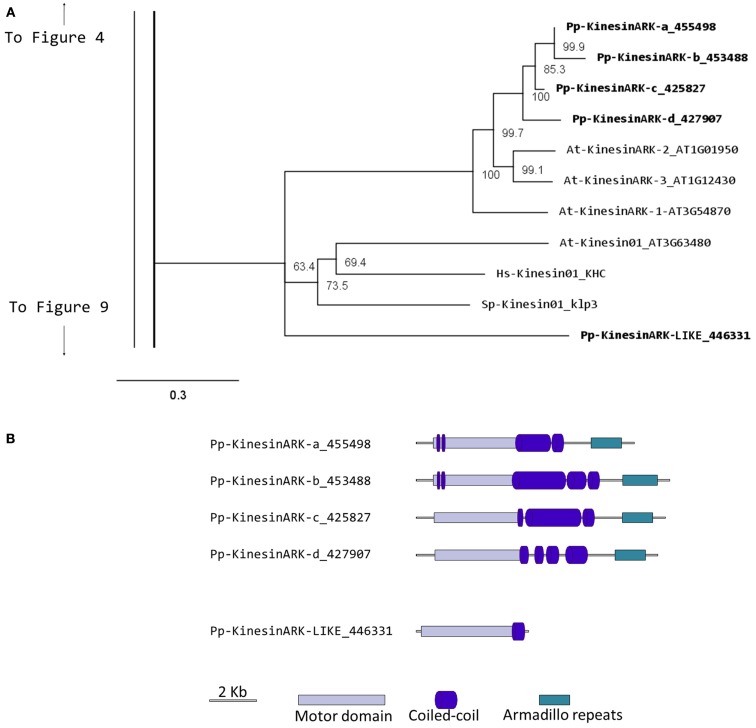
**(A)** Sub-region of the phylogenetic tree based on their motor domain showing kinesin 1s and armadillo repeat containing kinesins (ARKs). The amino acid sequences of the motor domain were aligned using ClustalW and the phylogenetic tree was constructed using the maximum likelihood method (PhyML) and a 1000 bootstrap resampling value. Numbers on the nodes show the statistical support of values above 50%. The scale shows the estimated branch length corresponding to the number of substitutions per site. The *Physcomitrella* numbers correspond to the Phypa number uniquely associated with each gene model (version 1.6) at cosmoss.org. **(B)** Gene models of kinesin 1s and ARKs. Schematic diagrams showing the structure and domain architecture of kinesin 1s and armadillo repeat containing kinesins (ARKs). Domains are indicated at the bottom of the diagrams. Armadillo repeats are comprised of a repeating sequence of forty-two amino acids that can form helices which upon repetition form a right handed super helix.

Phylogenetic analysis based on the motor domain indicates that there are five sequences in *Physcomitrella* related to the *Arabidopsis* ARK (Figure 1A). Four of these sequences are closely related to each other, forming a monophyletic group and their gene models show the presence of armadillo repeats (Figure 1B); we classified these as class I. The gene model for the fifth sequence is lacking the armadillo repeats that would confirm its identity as an armadillo repeat containing kinesin (Figure 1B); we tentatively classified this single kinesin as ARK-Like since the tree topology fails to confirm this kinesins as an ortholog of the lone At-kinesin 01. But it is intriguing that a very short gene model is also a landmark of this *Arabidopsis* kinesin 1 (Richardson et al., 2006).

It will be interesting to investigate if the participation of ARK in cell polarization that has been documented in *Arabidopsis* (Yang et al., 2007; Sakai et al., 2008), is also conserved in mosses, which provide an excellent model system to study cell polarization and tip growth. Furthermore, comparative analysis of loss of function phenotypes may help understand how this family of molecules functions in the cell.

### Kinesin 2

**Figure 2:**
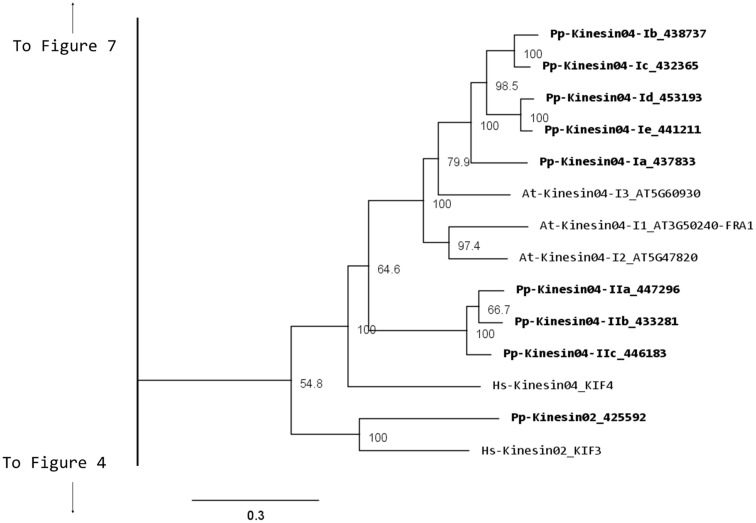
**Sub-region of the phylogenetic tree based on their motor domain showing kinesin 2s and kinesin 4s**. The amino acid sequences of the motor domain were aligned using ClustalW and the phylogenetic tree was constructed using the maximum likelihood method (PhyML) and a 1000 bootstrap resampling value. Numbers on the nodes show the statistical support of values above 50%. The scale shows the estimated branch length corresponding to the number of substitutions per site. The *Physcomitrella* numbers correspond to the Phypa number uniquely associated with each gene model (version 1.6) at cosmoss.org.

**Figure 3:**
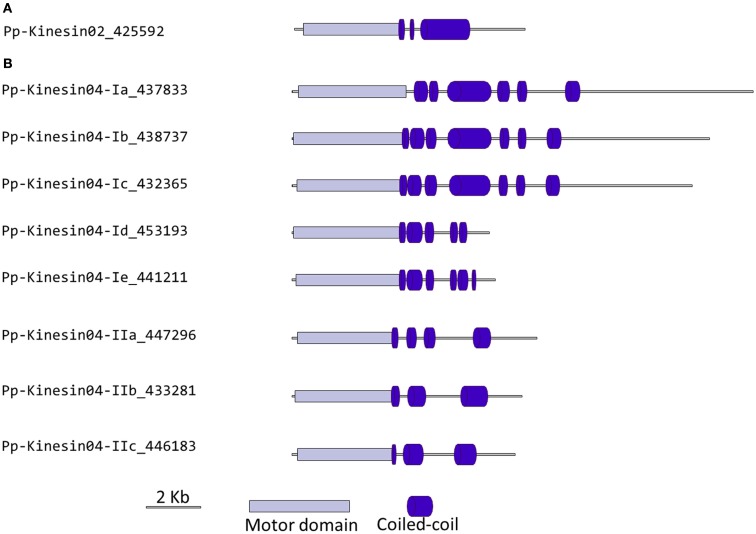
**Gene models of kinesin 2s and 4s**. Schematic diagrams showing the structure and domain architecture of **(A)** kinesin 2s and **(B)** kinesin 4s. Domains are indicated at the bottom of the diagrams.

Kinesin 2s have previously been shown to be involved in neuronal organelle transport (Yamazaki et al., 1995; Setou et al., 2000), meiosis in spermatogenesis (Wang et al., 2010), and intraflagellar transport (Sloboda and Howard, 2007). One of the common characteristics of kinesin 2s is their ability to create both homo and heterodimers (Rashid et al., 1995). However, in the case of *P. patens*, there is only one kinesin 2 present (Figure 2), and therefore it will only homodimerize, unless it can associate with a different kinesin. The protein itself is relatively short, containing two short coiled coils, and one large coiled coil (Figure 3A). In *Physcomitrella* this protein is likely to participate in the *de novo* formation of flagella during spermatogenesis. Consistently, kinesin 2s are absent in *Arabidopsis* and other seed plants which do not have flagella.

### Kinesin 4

This family is comprised of members that can bind to chromosomes in animals and are absent in budding and fission yeasts (Miki et al., 2005; Richardson et al., 2006). In animals, they are present in the nucleus as well as in the cytoplasm and they have been implicated in organelle and chromosomal transport (Miki et al., 2005). In plants, a member of this family was initially identified as a protein important for orienting cellulose microfibrils (FRA1). A mutation of the protein results in a fragile cell wall phenotype in *Arabidopsis* (Zhong et al., 2002); a similar mutant was also isolated in rice (Zhang et al., 2010). In addition, the rice kinesin was found to be nuclearly and cytoplasmically localized, and surprisingly it functions as a DNA binding protein important for gibberellin biosynthesis and cell elongation (Li et al., 2011). Single molecule analysis revealed that this molecule has unusually high processivity, suggesting a function in long-distance transport (Zhu and Dixit, 2011b).

Our phylogenetic analysis of kinesin 4s in *Physcomitrella* shows two well-defined classes (Figure 2), with class I clustering with the *Arabidopsis* kinesin 4s, including FRA1. Based on the available gene models, the five members of class I can be further subdivided into two classes, with Pp-kin04-Id and Pp-kin04-Ie having smaller C-terminal domains (Figure 3B). Class II is formed by three members, without counterparts in the *Arabidopsis* genome (Figure 2). This suggests the possibility that this class might carry out a function that is not present in seed plants. It would be interesting to determine whether the class I kinesin 4s have conserved a function in organizing the cell wall components in *Physcomitrella*, and whether both classes evolved similar or different functions.

It is interesting to note that there is an expanded collection of the kinesin 4s in *Physcomitrella* compared to *Arabidopsis*, the significance of this expansion remains to be elucidated.

### Kinesin 5

Kinesin 5s are tetrameric kinesins important for spindle organization and mitosis (Ferenz et al., 2010). In yeast, null mutants display division phenotypes such as delayed anaphase, larger cells, and abnormal spindle morphology (Hagan and Yanagida, 1992; Straight et al., 1998). This family spans multiple kingdoms as it is found in mammals, fungi, and plants (Miki et al., 2005; Richardson et al., 2006; Bannigan et al., 2008). Using a conditional loss of function approach, a similar but expanded function has been documented in plants, where one member of this family in *Arabidopsis* (AtKRP125c) was found to be important for spindle and cortical MT organization (Bannigan et al., 2007).

**Figure 4:**
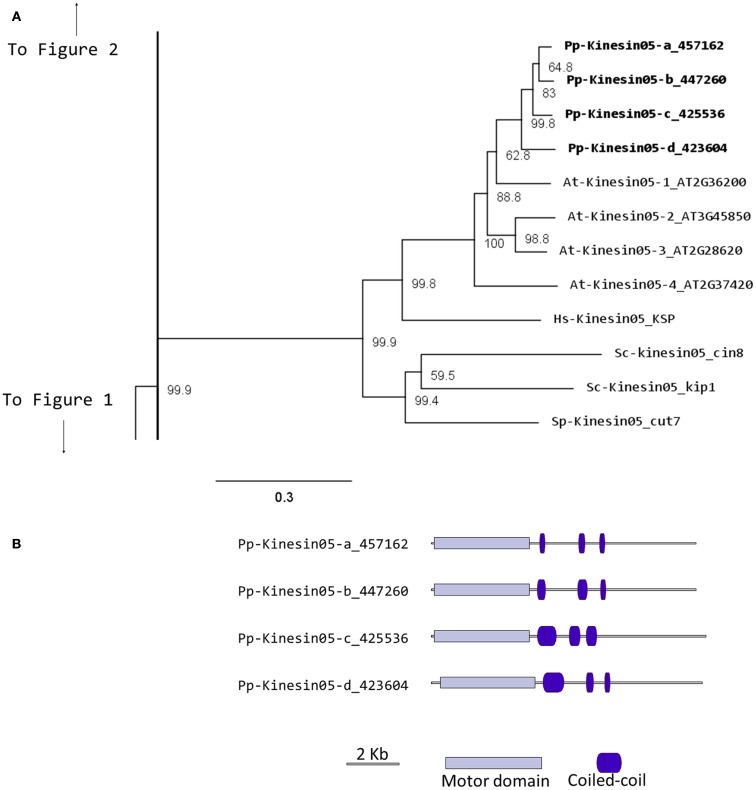
**(A)** Sub-region of the phylogenetic tree based on their motor domain showing kinesin 5s. The amino acid sequences of the motor domain were aligned using ClustalW and the phylogenetic tree was constructed using the maximum likelihood method (PhyML) and a 1000 bootstrap resampling value. Numbers on the nodes show the statistical support of values above 50%. The scale shows the estimated branch length corresponding to the number of substitutions per site. The *Physcomitrella* numbers correspond to the Phypa number uniquely associated with each gene model (version 1.6) at cosmoss.org. **(B)** Gene models of kinesin 5s. Schematic diagrams showing the structure and domain architecture of kinesin 5s. Domains are indicated at the bottom of the diagrams.

In *Physcomitrella*, there are four kinesin 5 members, which cluster as a monophyletic group (Figure 4A). Based on their gene models, they have a very similar structure (Figure 4B). We anticipate that these kinesins will perform similar functions to their *Arabidopsis*, animal, and fungal counterparts. Nevertheless, it is interesting that a mutation in only one of the four genes of *Arabidopsis* results in an altered growth phenotype (Bannigan et al., 2007), suggesting a degree of specialization in some of the kinesin 5s in *Arabidopsis*. Future functional analyses of the four moss isoforms will help clarify whether a similar type of specialization is present or absent in *Physcomitrella*.

### Kinesin 7

Members of the kinesin 7 family have been implicated in the transport of chromosomes and nuclear migration in animal and yeast (Miki et al., 2005). This family is greatly expanded in *Arabidopsis* with 14 members (Richardson et al., 2006; Zhu and Dixit, 2011a). Functional analysis of some of its members has shown a participation in cell division. For example, loss of function of AtNACK1 and AtNACK2 results in inhibition of cytokinesis (Tanaka et al., 2004; Takahashi et al., 2010); a similar phenotype was found in rice, when the expression of the single OsNACK gene is reduced in a leaky mutant (Sazuka et al., 2005). Other members of this family have a mitochondrial signaling sequence, but their function has yet to be investigated (Itoh et al., 2001).

**Figure 5:**
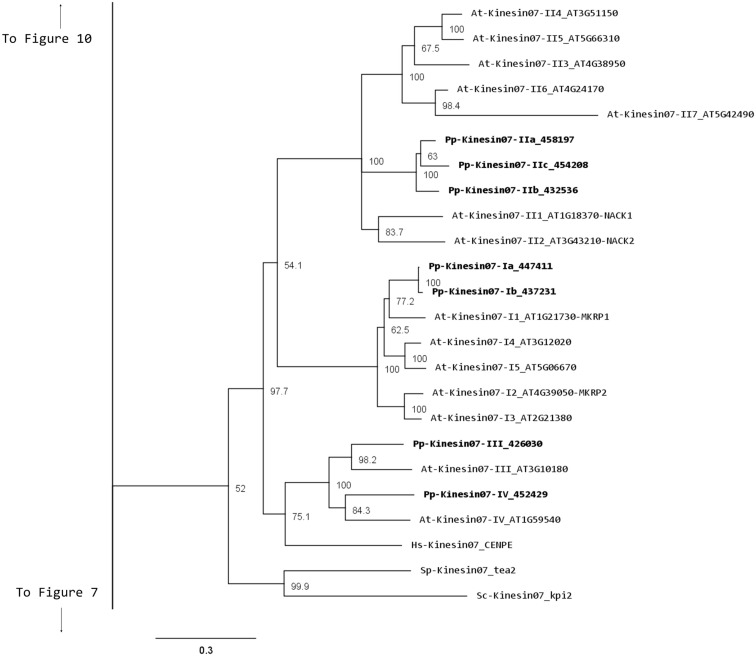
**Sub-region of the phylogenetic tree based on their motor domain showing kinesin 7s**. The amino acid sequences of the motor domain were aligned using ClustalW and the phylogenetic tree was constructed using the maximum likelihood method (PhyML) and a 1000 bootstrap resampling value. Numbers on the nodes show the statistical support of values above 50%. The scale shows the estimated branch length corresponding to the number of substitutions per site. The *Physcomitrella* numbers correspond to the Phypa number uniquely associated with each gene model (version 1.6) at cosmoss.org.

Our phylogenetic analysis shows a smaller size for this family in *Physcomitrella* with seven members compared with *Arabidopsis* (Figure 5); nevertheless the classes found seem to be conserved between species. We identified four classes, with class I containing the MKRP-related kinesins, that could be associated with organelles (Itoh et al., 2001). *Physcomitrella* has only two representatives for this class, compared with five for *Arabidopsis*. Class II has three representatives in *Physcomitrella*, compared with seven in *Arabidopsis*. The moss class II kinesin 7s seem to represent an independent monophyletic group with no specific clustering to the *Arabidopsis* subgroups. Unfortunately this makes it difficult to clearly define a functional ortholog to the well-characterized NACK kinesins (Tanaka et al., 2004; Takahashi et al., 2010; Sasabe et al., 2011), and further functional analysis will be needed to determine whether the moss class II kinesin 7s are also involved in cytokinesis.

**Figure 6:**
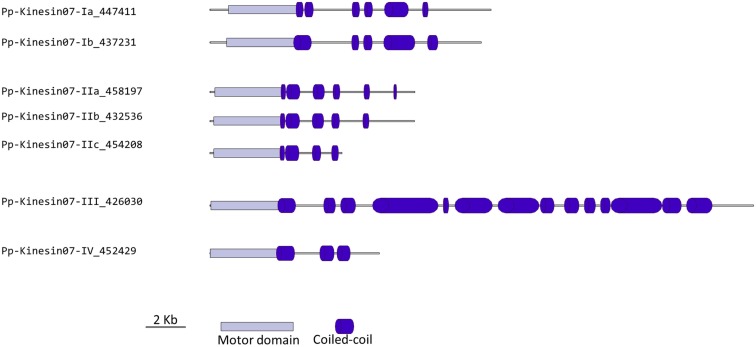
**Gene models of kinesin 7s**. Schematic diagrams showing the structure and domain architecture of kinesin 7s. Domains are indicated at the bottom of the diagrams.

Classes III and IV are closely related at the motor domain level (Figure 5), but their C-terminal domains are very different, with class III containing a much longer coiled coil rich domain (Figure 6). Interestingly, orthologs exist for both classes in *Arabidopsis*, an indication that the common ancestor of mosses and vascular plants contained these two classes. These classes are more closely related to CENPE and may share some of its function on kinetochore capture (Weaver et al., 2003).

### Kinesin 8

**Figure 7:**
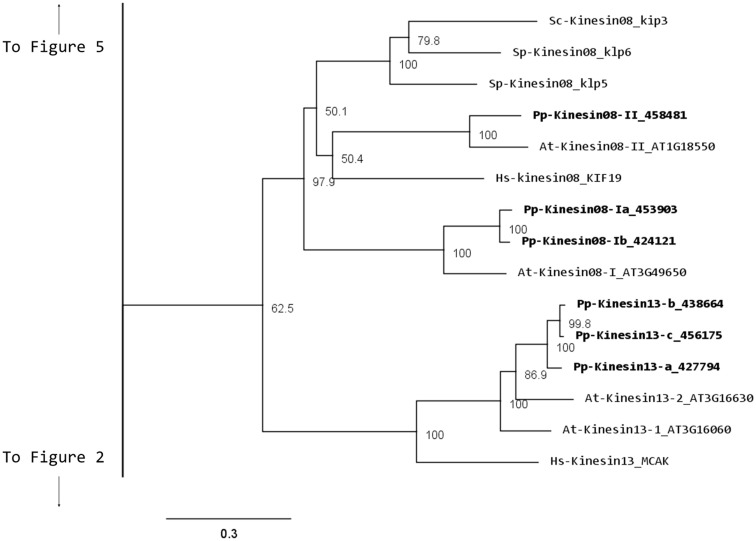
**Sub-region of the phylogenetic tree based on their motor domain showing kinesin 8s and kinesin 13s**. The amino acid sequences of the motor domain were aligned using ClustalW and the phylogenetic tree was constructed using the maximum likelihood method (PhyML) and a 1000 bootstrap resampling value. Numbers on the nodes show the statistical support of values above 50%. The scale shows the estimated branch length corresponding to the number of substitutions per site. The *Physcomitrella* numbers correspond to the Phypa number uniquely associated with each gene model (version 1.6) at cosmoss.org.

**Figure 8:**
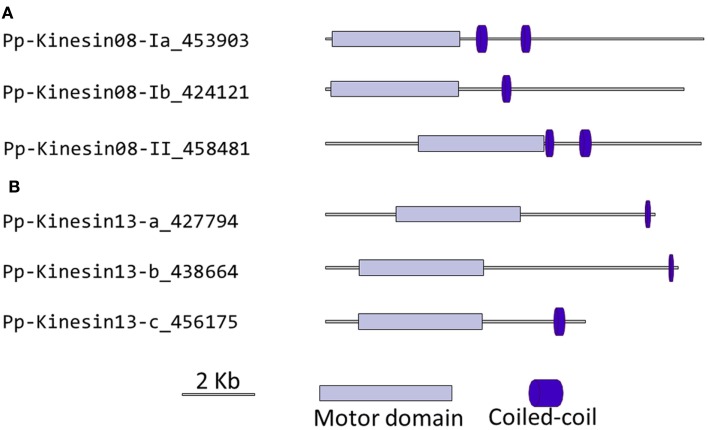
**Gene models of (A) kinesin 8s and (B) kinesin 13s**. Schematic diagrams showing the structure and domain architecture of kinesin 8s and 13s. Domains are indicated at the bottom of the diagrams.

Although nothing is known about kinesin 8s in plant systems, significant research has been conducted on these kinesins in animal and fungi. Some of the group’s functions include mitochondrial transport in *Drosophila*, mitotic chromosome segregation in yeast, and MT destabilization in humans (Miki et al., 2005; Peters et al., 2010). Our phylogenetic analysis indicates that there are two kinesin 8 classes for both moss and *Arabidopsis* (Figure 7). Of these two, class I contains a single *Arabidopsis* kinesin and two moss kinesins; class II contains a single moss and *Arabidopsis* kinesin. The gene model for the moss class II kinesin shows an extended N-terminal domain (Figure 8A). Because of their similarity to other kinesin 8 members from animals and fungi, we anticipate these kinesins will have a conserved function. Nevertheless, the existence of two ortholog genes in plants suggests that diversification of function was already present, to some degree, in the last common ancestor of bryophytes and vascular plants.

### Kinesin 9

**Figure 9:**
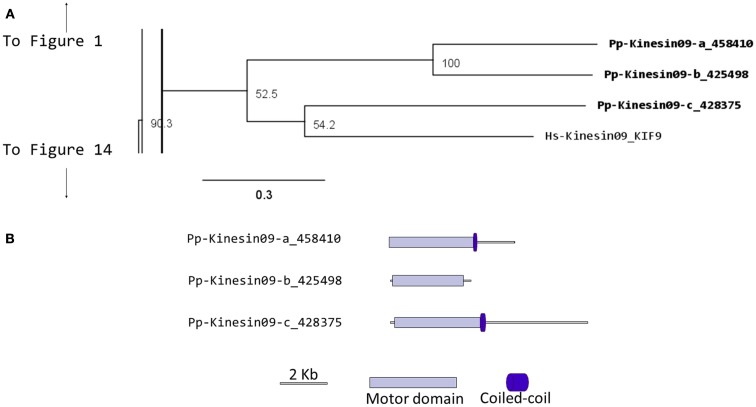
**(A)** Sub-region of the phylogenetic tree based on their motor domain showing kinesin 9s. The amino acid sequences of the motor domain were aligned using ClustalW and the phylogenetic tree was constructed using the maximum likelihood method (PhyML) and a 1000 bootstrap resampling value. Numbers on the nodes show the statistical support of values above 50%. The scale shows the estimated branch length corresponding to the number of substitutions per site. The *Physcomitrella* numbers correspond to the Phypa number uniquely associated with each gene model (version 1.6) at cosmoss.org. **(B)** Gene models of kinesin 9s. Schematic diagrams showing the structure and domain architecture of kinesin 9s. Domains are indicated at the bottom of the diagrams.

Kinesin 9s have been implicated in flagella regulation, structure, and construction (Bernstein et al., 1994; Yokoyama et al., 2004; Demonchy et al., 2009), and consistent with this function, they are absent in flowering plants, yeast, and invertebrates (Richardson et al., 2006). Our phylogenetic analysis identified three kinesin 9s in *Physcomitrella* (Figure 9A). Two of the three gene models found in the cosmoss.org database (Pp-Kinesin09-b and c) seem to be inaccurate because of some abnormal insertions and gaps are present when compared to other kinesin 9 sequences. The genomic sequences corresponding to the questionable regions were examined in more detailed and it was found that some exons are not present in the latest proteome version in cosmoss.org (version 1.6) and that some introns were incorrectly spliced (Figure 9B and Figure S1 in Supplementary Material). We were not able to determine if additional problems exist in the gene models of the regions after the motor domain, which due to reduced conservation are harder to identify and their detailed description is beyond the scope of this manuscript. Similarly to kinesin 2 (Sloboda and Howard, 2007), we anticipate kinesin 9s will participate in the *de novo* assembly of flagella during spermatogenesis in moss.

### Kinesin 10

The kinesin 10 family members are commonly referred as “Kid” in human (Tokai et al., 1996) and “KIF 22” in mouse (Yang et al., 1997). They have been suggested to be involved in spindle formation and chromosome movement (Miki et al., 2005). It is notable that members of the kinesin 10 family, which are present in *Arabidopsis* (two members) are absent in *Physcomitrella* (Figure 14). Although PAKRP2 has been sometimes grouped in the kinesin 10 family (Richardson et al., 2006; Zhu and Dixit, 2011a), it is more appropriate to be classified as an orphan kinesin based on our analysis (Figure 14). A detailed discussion about this classification can be found at the section for orphan kinesins below. The presence of orthologs of the *Arabidopsis* kinesin 10s in other basal plant species may provide clues about essential developmental processes present in a common ancestor but lost in mosses.

### Kinesin 12

In animals, kinesin 12s have been implicated in bipolar spindle assembly (Rogers et al., 2000; Tanenbaum et al., 2009) and neuron development and axon growth (Liu et al., 1996; Buster et al., 2003). In plants, kinesin 12s have been found to be involved in phragmoplast organization and orientation (Lee and Liu, 2000; Pan et al., 2004; Muller et al., 2006). In general, kinesin 12s have an N-terminus head with a long C-terminus tail abundant in coiled coils (Miki et al., 2005).

**Figure 10:**
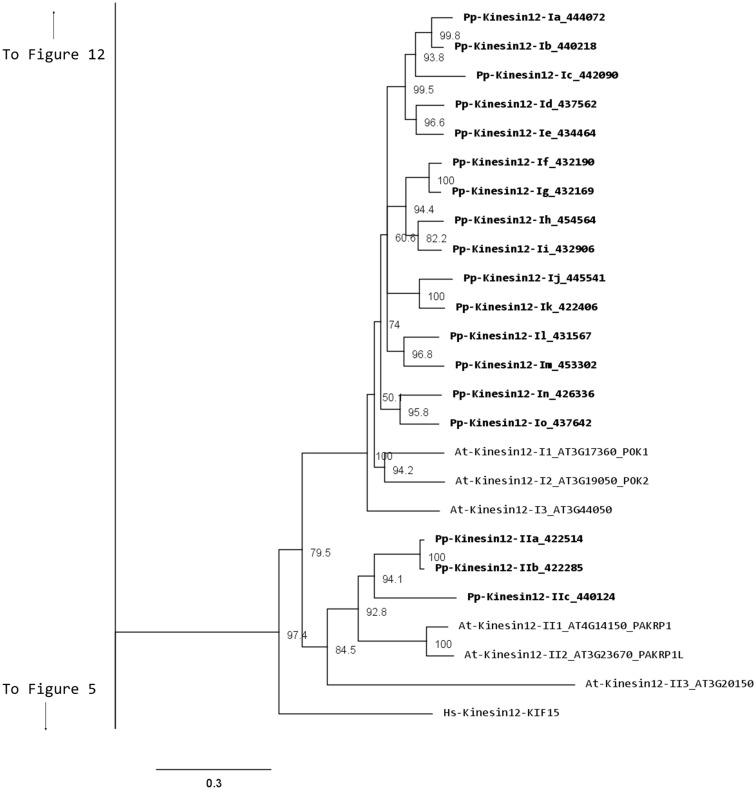
**Sub-region of the phylogenetic tree based on their motor domain showing kinesin 12s**. The amino acid sequences of the motor domain were aligned using ClustalW and the phylogenetic tree was constructed using the maximum likelihood method (PhyML) and a 1000 bootstrap resampling value. Numbers on the nodes show the statistical support of values above 50%. The scale shows the estimated branch length corresponding to the number of substitutions per site. The *Physcomitrella* numbers correspond to the Phypa number uniquely associated with each gene model (version 1.6) at cosmoss.org.

**Figure 11:**
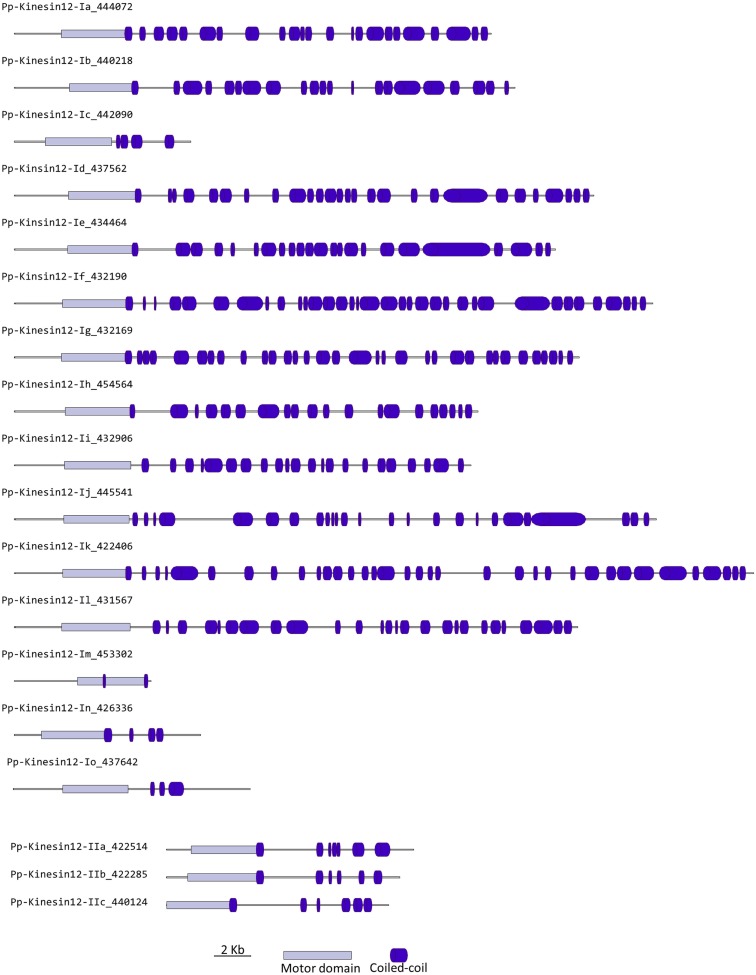
**Gene models of kinesin 12s**. Schematic diagrams showing the structure and domain architecture of kinesin 12s. Domains are indicated at the bottom of the diagrams.

Our phylogenetic analysis shows two classes of kinesins 12s; class I kinesin 12s, which are related to the phragmoplast orienting kinesins or POKs (Muller et al., 2006), and class II kinesin 12s, which are related to the phragmoplast-associated kinesin 1s or PAKRP1s (Figure 10). We found a surprisingly large number of class I kinesin 12s in *Physcomitrella*: a total of 18 genes, compared with only three in *Arabidopsis*. Some of the gene models corresponding to regions after the motor domain seem to be incomplete, but the majority of the class I sequences show long C-terminal domains with abundant coiled coils (Figure 11). The significance of this large number of kinesins is not understood and presents a challenging problem due to the likelihood of functional redundancy between its members. Nevertheless, due to their similarity to *Arabidopsis* POKs, these proteins are probably important for phragmoplast orientation. In contrast to the large number of class I kinesins 12s, there are only three class II kinesin 12s in *Physcomitrella*, forming a monophyletic group (Figure 10). The gene models for these kinesins show very similar C-terminal structures with abundant coiled coil structures, but not of the large magnitude of the ones present in class I (Figure 11). Again, we anticipate that these kinesins will play a similar role in phragmoplast organization as that of their *Arabidopsis* counterparts.

### Kinesin 13

Similar to kinesin 8s, the animal kinesin 13s have been reported to destabilize MT and to function in intracellular transport (Miki et al., 2005). Although both plant and animal kinesin 13s share similar motor domain sequences, the kinesin 13s in plants do not have a lysine-rich neck domain present in animal kinesin 13s (Lee and Liu, 2004). The absence of this structural motif suggests that the plant kinesins may have a different function than their animal counterparts (Lee and Liu, 2004). Consistently, the *Arabidopsis* kinesin-13-2, which has been shown to be associated with Golgi stacks, provides further evidence that plant kinesin 13s may differ from animal kinesin 13s in functionality (Lu et al., 2005).

Our phylogenetic analysis shows that in *Arabidopsis* there are two kinesin 13s while in *Physcomitrella*, there are three kinesin 13 members, which cluster as a monophyletic group (Figure 7). Based on their gene models, the moss kinesin 9s have a very similar structure (Figure 8B). Interestingly, for the three moss kinesin 9s, the sole coiled coil region is located at the extreme C-terminus of the molecule away from the motor domain; while in most of the kinesins, the coiled coil regions are generally closer to the motor domain (Figure 8B). It would be interesting to determine whether this structural characteristic is important for the function of these kinesins. The *Arabidopsis* kinesin-13-2 is closely related to those found in moss (Figure 7), which suggests possible functional conservation between the *Arabidopsis* kinesin-13-2 and all the moss kinesin 13s.

### Kinesin 14

**Figure 12:**
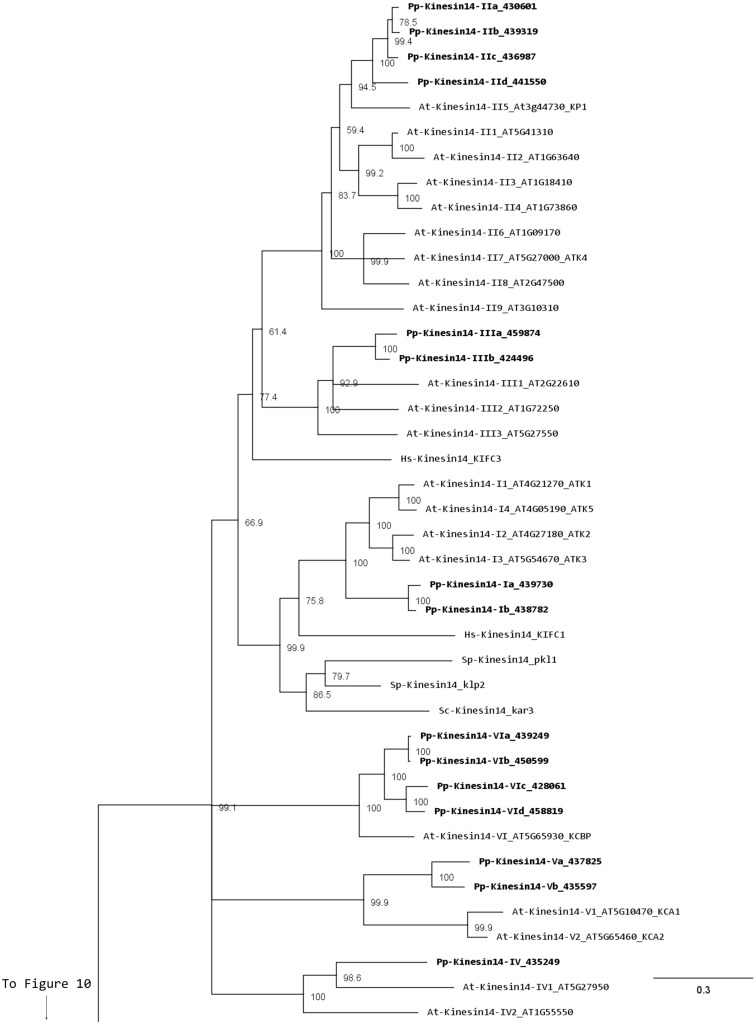
**Sub-region of the phylogenetic tree based on their motor domain showing kinesin 14s**. The amino acid sequences of the motor domain were aligned using ClustalW and the phylogenetic tree was constructed using the maximum likelihood method (PhyML) and a 1000 bootstrap resampling value. Numbers on the nodes show the statistical support of values above 50%. The scale shows the estimated branch length corresponding to the number of substitutions per site. The *Physcomitrella* numbers correspond to the Phypa number uniquely associated with each gene model (version 1.6) at cosmoss.org.

Members of this family have been associated with functions in re-arrangement of the MT arrays at various stages of the cell cycle as well as in organelle transport (Miki et al., 2005; Richardson et al., 2006; Zhu and Dixit, 2011a). Kinesin 14s were initially divided in two groups, kinesin 14A and kinesin 14B, according to their structure and function (Miki et al., 2005). However, this family is vastly expanded in plants with 21 and 15 members in *Arabidopsis* and *Physcomitrella*, respectively, compared to 4 in humans (Richardson et al., 2006). In addition, the fact that plant kinesins 14 display some specific structural motifs prompted us to propose a new classification for the plant kinesin 14s, divided in six different classes (Figure 12).

**Figure 13:**
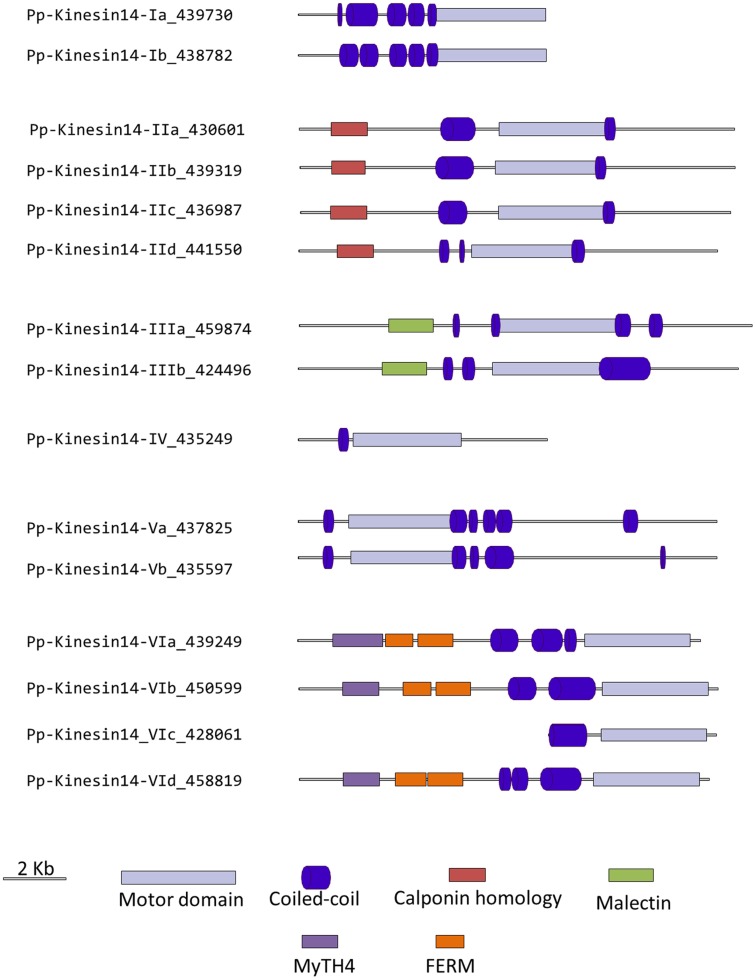
**Gene models of kinesin 14s**. Schematic diagrams showing the structure and domain architecture of kinesin14s. Domains are indicated at the bottom of the diagrams. Calponin homology domains mediate interaction with F-actin; Malectin domains allow binding to carbohydrate such as di-glucose; and Myosin Tail Homology domain 4 (MyTH4) and FERM domains (motif named after proteins that contains it: 4.1 protein, Ezrin, Radixin, Moesin) also called talin-like region, are known to bind microtubules.

Class I kinesin 14s are related to KIFC1 which is associated with the nuclear membrane in mammalian cells and is important for acrosome biogenesis and possibly for vesicle transport (Yang and Sperry, 2003; Yang et al., 2006; Nath et al., 2007), and to Kar3p which is essential for nuclear fusion during mating in *S. cerevisiae*, by mediating MT sliding (Meluh and Rose, 1990). Among the four homologs present in *Arabidopsis* (Figure 12), ATK1 and ATK5 have been well studied. They share similar functions during mitosis by controlling the MT organization at the cortex, the preprophase band, the spindle, and the phragmoplast, and ATK1 also play a major role in male meiosis (Liu et al., 1996; Chen et al., 2002; Marcus et al., 2003; Ambrose et al., 2005; Ambrose and Cyr, 2007). Interestingly, ATK5 possesses a second, ATP independent, MT binding site on its N-terminal region, that could be important for bundling, a property already reported for the kinesin Ncd in *Drosophila* (Furuta and Toyoshima, 2008). Class I in *Physcomitrella* is composed of two members (Figure 12), which contain a C-terminal motor domain (Figure 13), and therefore, similar to ATK5 (Ambrose et al., 2005), are likely to be minus-end directed motors.

Members of classes II and III are related to KIFC3, which is involved in Golgi positioning and integration in mouse (Xu et al., 2002). Class II is the largest class with eight members in *Arabidopsis* and four in *Physcomitrella* (Figure 12). In *Arabidopsis*, AtKP1 has been shown to organize the cortical MT array (Ni et al., 2005; Yang et al., 2011), and to regulate mitochondrial functions (Yang et al., 2011). ATK4 and homologs in cotton and rice, possess a Calponin Homology (CH) domain that mediates interaction with F-actin (Tamura et al., 1999; Preuss et al., 2004; Frey et al., 2009; Xu et al., 2009), and could be important for regulating the motor activity and coordinating the activities of MT and actin microfilaments during premitotic nuclear migration (Umezu et al., 2011). Interestingly, the four members of this class in *Physcomitrella* also contain the CH domain (Figure 13), strongly suggesting that the binding to actin microfilaments is conserved in moss.

Class III has three representatives in *Arabidopsis* and two in *Physcomitrella* (Figure 12). However, the function of these kinesins still remains unknown. Intriguingly, these kinesins possess a malectin domain (Figure 13), which allows binding to carbohydrate such as di-glucose (Schallus et al., 2008). This domain has been identified in malectins, which are conserved proteins of the endoplasmic reticulum in animals and involved in protein *N*-glycosylation (Schallus et al., 2008), as well as in plasma membrane-located leucin-rich repeat receptor kinases such as FERONIA in *Arabidopsis*, where it is thought to regulate cell growth in response to cell wall changes (Zou et al., 2011). The functional significance of the malectin domains for kinesins is unclear and further investigation will be needed to decipher the role of class III kinesin 14s in plants.

Class IV kinesin 14s are plant specific. The homolog in tobacco, TBK5, is thought to function in relocating and gathering newly formed MTs and/or MTs nucleating units (Goto and Asada, 2007). *Physcomitrella* possesses only one member compared to two in *Arabidopsis* (Figure 12), which makes it a great system to gain more insight into the function(s) fulfilled by these kinesins in plants.

Class V kinesin 14s KAC1 and KAC2 have been recently identified in *Arabidopsis* in a genetic screen for chloroplast movement in response to light intensity changes (Suetsugu et al., 2010). Interestingly, they show no MT binding activity or detectable ATPase activity. Instead, they are thought to interact with actin microfilaments and mediate chloroplast movement in an actin-dependent manner. However, the precise mechanism by which they regulate chloroplast movement still needs to be determined. *Physcomitrella* also contains two members of this class (Figure 12) and whether or not they interact with MTs and/or actin filaments to move chloroplasts is not known. It will be interesting to investigate whether these two members of the class V kinesin 14 have similar functions as their *Arabidopsis* homologs. We want to note that gene model corresponding to the N-terminal sequence for kinesin 14-Vb is incorrect in Phytozome due to an incorrect prediction of a splicing site. This does not affect the motor domain sequence that was used for our phylogenetic tree. We provide in the supplementary material what we believe is the correct protein sequence for this molecule (Figure S1 in Supplementary Material).

Surprisingly, class VI encompasses only one member in *Arabidopsis*, KCBP, compared to four in *Physcomitrella* (Figure 12). KCBP, which contains a calmodulin-binding domain, participates in cortical MT organization (Oppenheimer et al., 1997), and is involved in the different stages of mitosis by regulating bundling and sliding of MT (Bowser and Reddy, 1997; Vos et al., 2000). The four moss homologs contain a myosin tail homology domain 4 (MyTH4) and two FERM domains (motif named after proteins that contains it: 4.1 protein, Ezrin, Radixin, Moesin) also called talin-like region (Figure 13), which are known to bind MTs (Narasimhulu et al., 1997). Therefore, moss class VI kinesin 14s are likely to function in cross-linking or bundling of MTs.

### Orphan kinesins

**Figure 14:**
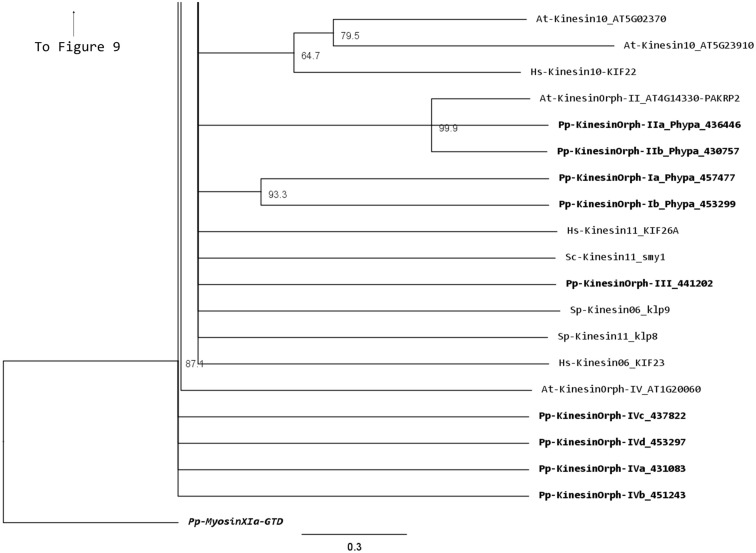
**Sub-region of the phylogenetic tree based on their motor domain showing kinesin 6s, 10s, 11s, and orphan kinesins**. The amino acid sequences of the motor domain were aligned using ClustalW and the phylogenetic tree was constructed using the maximum likelihood method (PhyML) and a 1000 bootstrap resampling value. Numbers on the nodes show the statistical support of values above 50%. The scale shows the estimated branch length corresponding to the number of substitutions per site. The *Physcomitrella* numbers correspond to the Phypa number uniquely associated with each gene model (version 1.6) at cosmoss.org.

**Figure 15:**
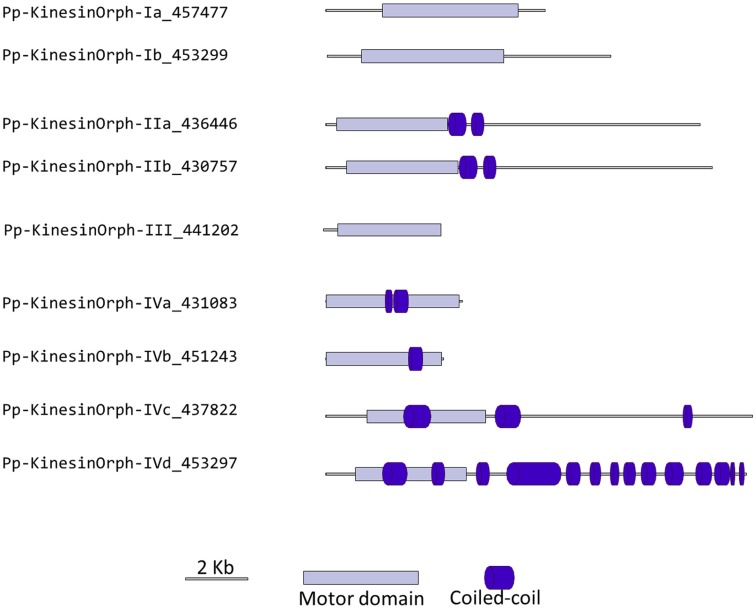
**Gene models of orphan kinesins**. Schematic diagrams showing the structure and domain architecture of orphan kinesins. Domains are indicated at the bottom of the diagrams.

We have grouped the remainder moss kinesins into four classes based on the similarity of their motor domain. Class I is composed of two related kinesins with no homologs in *Arabidopsis* or animals (Figure 14). The gene models for the region outside of the motor domain may not be well predicted due to limited transcript sequence information, so it is difficult to deduce any specific function from the available gene model sequence (Figure 15). A similar situation is present for the single member of class III (Figures 14 and 15). Due to the small number of members in these classes it should be relatively simple to evaluate their function using the various loss of function techniques available in *Physcomitrella*. However, it is also relevant to mention that at this point it is hard to rule out the possibility that these genes might be pseudogenes.

The class II orphan kinesins are composed of a pair of kinesins (KINID1a and KINID1b) that have been shown to be important for interdigitation of phragmoplast MTs and cell plate expansion in moss (Hiwatashi et al., 2008). These kinesins are orthologs of PAKRP2 from *Arabidopsis*, which is predicted to function in the transport of Golgi-derived vesicles in the phragmoplast (Lee et al., 2001); nevertheless a conserved function between the moss and *Arabidopsis* orthologs has so far not been established (Hiwatashi et al., 2008). In previous analysis, where PAKRP2 was classified as a kinesin 10, the clade that PAKRP2 belonged to was parallel with other kinesin families as well as the second clade of Kinesin 10. In addition, that particular clade was not resolved in three of the four methods used to build the tree and had a low score in the methods that resolved the clade (Richardson et al., 2006). Based on our analysis, we suggest an orphan classification for PAKRP2 because it groups with the moss orthologs with a high bootstrap score, but not with *bona fide* kinesin 10s. Furthermore, the class II orphan kinesins have much longer C-terminal domains when compared with kinesin 10s. It is therefore more appropriate for these kinesins to be classified as orphan kinesins.

There is only one class III kinesin found in *Physcomitrella*. This kinesin is highly divergent since its relationship cannot be resolved between kinesin 6s and kinesin 11s from human and yeasts in our phylogenetic tree (Figure 14). Whether this kinesin will have conserved function to kinesin 6 or 11 is not clear form our tree, but kinesin 6s and 11s are known to be highly divergent (Miki et al., 2005). In addition, the lacking of gene model information on the C-terminal after the motor domain casts shadow on the possibility that it might also be a pseudogene (Figure 15).

The final group of kinesins is class IV; these kinesins have the most divergent motor domains and tend to cluster with kinesin 11 members from animals and yeasts (Figure 14). In yeast, kinesin 11 or Smy1p does not bind to MTs and it seems to regulate myosin V function (Lillie and Brown, 1998; Beningo et al., 2000). The four class IV members in *Physcomitrella* have coiled coils in their head domains (Figure 15), suggesting a non-functional motor similar to yeast. Therefore these four kinesins are classified as class IV of the orphan kinesins. Besides the relatively low homology in the motor domains, there is little additional similarity in the rest of the molecules from this class (Figure 15). Analogous to class I and III kinesins, it will be important to determine their phylogenetic distribution and conservation in other species, but a detailed loss of function and biochemical analyzes will be required to determine their function.

## Conflict of Interest Statement

The authors declare that the research was conducted in the absence of any commercial or financial relationships that could be construed as a potential conflict of interest.

## Supplementary Material

The Supplementary Material for this article can be found online at http://www.frontiersin.org/Plant_Cell_Biology/10.3389/fpls.2012.00230/abstract

Supplementary Figure S1 **Updated sequences for Pp-Kinesin09-b, Pp-Kinesin09-c, and Pp-Kinesin14-Vb**. The bold sequences indicate exons absent from the Phytozome database. These sequences were updated from the genomic sequence information available on JGI (www.jgi.doe.gov) by aligning representative kinesins from multiple species.
