# Supplementary material for: Phylogenetic Analysis of the Kinesin Superfamily from Physcomitrella
Source: Front Plant Sci. 2012 Oct 16;3:230. doi: 10.3389/fpls.2012.00230 (PMC3472504; doi:10.3389/fpls.2012.00230)
Supplement: Supplementary Figure S1 — Updated sequences for Pp-Kinesin09-b, Pp-Kinesin09-c, and Pp-Kinesin14-Vb. The bold sequences indicate exons absent from the Phytozome database. These sequences were updated from the genomic sequence information available on JGI (www.jgi.doe.gov) by aligning representative kinesins from multiple species. [file 31423_Vidali_DataSheet1.PDF]

Supplementary sequences:

>Pp-Kinesin09-b_425498_Motor_Domain

SRIRVYLRLRPSVKPSPAINIESETHRVLIDVEKSIGGGPPKAYVNQIVFNVDNIVQ**TTNQQTMYELCAKASVDEFLKGYN**
STIMSYGQVGAGKTFTMTGDMKVYVHRGIIPRAIQQIFEEKEAKPEAGIVVHMSYMEIYQEGLYDLLQKRRDDLMIIE
DNQLLNVRGLAKVRVETETEALKWFQEGEKSRSFGNHFLNSLSSRSHTILTFYMERRVARVSTQLALQVAKLNLVDLAG
VERLKKTKGDTGSLMRKEACINNKTLSFLEQTIFALRLKKAHIPFRHSKVTTLLKESLGNNHKTVFMVCAWPEEYFLDETI
GALRFAQRVKYLKIFQVTHKKPDCADTT

>Pp-Kinesin09-c_428375_Motor_Domain

MQLFVSSTKFSAMGAGFDSTIDIYLRVRPISSGAKAVLELNQEEGRVTWTIPRHVSLGLANHQREHFTFKFTGLFDMESK
QDEVFQKVAHKVVIGSLDGYNGTIFAYGQTGSGKTYTITGGSERYVDRGIIPRTISLIFSEIAERSEYAYTLHFSYMEVYNET
GYDLLNPDHETKALEDLPKDFILANEPIIANYQFANAFRVATEEEALNLVFVGDTNRIISSTPMNMASSRSHCIFTAHILAC
KVGEETVRKSKLHLVDLAGSERVWKTGVDGQ**ILREAKYINLSLHYLEQ**VIVALQEKFQGKMRTHIPYRNSMMTSVLRD
SIGGNCLTVMIATVTIAQDQLPETISTCRFAQRVAMISNQVTLNEEVDPNLLI

>Pp-Kinesin14_Vb_435597_Full_Length

MGDAKGVRNSWGGGLPSYRQFDADDEPRVREAPVYVPQSPSLTGRVPQSPSLAGRVPQSPSLAGRRHSISAVQFP
DTPKQKSLQSPTFVSKVLKVKDRLSSAREECIELRQEASDLQEYSNAKIERVTRYLGVLAEKARRLDEVALDSESRVTPL
KKEKKKLFNELVSAKGNVRVYCRARPQFEDEGPSSTTYPDDFTLRLNSNVTAAPNKDFELDRIYGPHISQADIFQDLQPL
VQSALDGFNVSIFAYGQTGAGKTFTMEGPSHDRGLYYRVLEELFDLVNSEATPTSSTSFFVTMFELYNEQVRDLLKAPD
NRGASTVLFGEPGRGVELVDERLDSPSGFARIFKFGKQMRANVDGVKFDRSSRSHLVVTIHIHSSDSLTGEEHYSKLSMV
DLAGSERLNKAEANGDRLTESLHINKSLSALGDVLSALTTKKDYIPYDHSKLTELLYDSLGGDSKAVLIANVNPSNAEVQE
TIATLNFASRARSAEISLGNRDTIKKWRDMASEARKELYEKEKEATEALGEVMQLKRALKESDDQCLLLFGEVQKAWKL
ASSLQADLTSHESYINKLQLENDRLSEQSIRDKEQYTNVLTQLTTFTTREEQYQSQIKERSARNEALEVRVQVLEQQLNEA
RVAAARTLPARPDNSAELQRLREETENALDMNQKLEEELSKRDELIERLHQENEKLFERLTDRSMTTISSPRVSSTPKIPR
AESRGMDDFNLDGGFVSATAVPGSPDMRSSSSMRESAGPPSSPGGSGALLKYGSGESVKSTPAGEYLTAALMDFNPA
QYESDAAIADGANKLLMLVLAAVIKAGASREHEMLAEIQGAVFGFLHKMENLLVMDTMLVSRVRILYIRSLLSRAPELQ
SLKVPPVERFLEKAGSGSATGSGSGRSSRNSSLGSSPQRSPAHRNKGADDYGPGFKVSLRQEKRSKFSSLVSKLMGNGD
QDNGRPHVTEGKLKETTEEARAFAIGNKGLASLFVHTPAGELQRQIRGWLAENFDFLSLTGEESIGGVTSHLELLSTAILD
GWMSGLGVPAQPSTDALGQLLSDYTSMVYTRQLQHLTDVAATLATEEAEDAAQVTKLRSALESVEHKRRKVLMQMR
TNVALLSKDDATSPTRSSSMDTENVRIASLMSLDDFFKQAEEIYRDAPHGTTTVNKKMTYLRRLDALEERMSTLLSIDQP
CANKCIMDARKFVESLEEQQVVSGGRHSRSRTMDELDASGNIGQWDEAMNNGVESEVVQWSVLQFNNGSATPFVI
KCGATSNLELVVKAQAKMQEKNGKEIVAVVPVPSVLDGLSVETIRQTISHLPESFLQLAMARTADGTRARYTRLYKTLAI
RVPGLKHVVEELEETSMSK

***Note: The sequence in bold is absent in Phytozome.**
